# A Rare Case of Metastatic Adenoid Cystic Carcinoma in the Liver: A Case Report

**DOI:** 10.7759/cureus.92595

**Published:** 2025-09-17

**Authors:** Sohaib Khalid, Iram Khan, Urfa Shafi, Muhammad Atique

**Affiliations:** 1 Histopathology, Pakistan Kidney and Liver Institute and Research Center, Lahore, PAK; 2 Histopathology, Chughtai Institute of Pathology, Lahore, PAK

**Keywords:** adenoid cystic, liver lesion, metastasis, rare case, submandibular swelling

## Abstract

Adenoid cystic carcinoma (ACC) is among the most common primary malignant tumors of the salivary glands. The parotid, submandibular, and minor salivary glands are the predominant sites. Clinical presentation includes a slow-growing mass, often accompanied by pain and paresthesias. Distant spread of the tumor is common - most frequently to the lungs, followed by the bones and other sites such as the liver, brain, and soft tissue. Metastatic ACC involving the liver is exceedingly rare. Given the uncommon occurrence of hepatic metastasis in ACC, we present the case of a 54-year-old female with metastatic involvement of both the lungs and liver.

## Introduction

Adenoid cystic carcinoma (ACC) is one of the rare primary malignancies of the salivary glands. The parotid, submandibular, and minor salivary glands are the predominant sites [1]. It typically follows a long, indolent course, with a high rate of distant metastasis. Distant spread of the tumor is common - mostly to the lungs, followed by the bones, and then to sites such as the liver and brain [1,2]. The most common mode of metastasis is hematogenous [3]. Due to the rare occurrence of this cancer in the liver, we report a case of a 55-year-old female who presented with decompensated chronic liver disease and was diagnosed with metastatic ACC involving the lungs and liver.

## Case presentation

On September 9, 2024, a 55-year-old female presented to the outpatient clinic at the Pakistan Kidney and Liver Institute (PKLI), Lahore, Pakistan, with complaints of abdominal pain and distention. Based on her history, clinical examination, and laboratory investigations, she was diagnosed with hepatitis C with decompensated liver disease. She also reported a history of right submandibular swelling for the past 10 years and painful cervical lymphadenopathy for the last one year. Fine-needle aspiration cytology (FNAC) of the cervical swelling, performed at an outside center, was suggestive of a benign salivary gland neoplasm, and no further management was done for these swellings. Computed tomography (CT) triphasic scan of the chest and abdomen revealed multiple lesions in the lungs and liver. Subsequently, a liver lesion biopsy was performed and sent to us for histopathological examination.

Radiology

**Figure 1:**
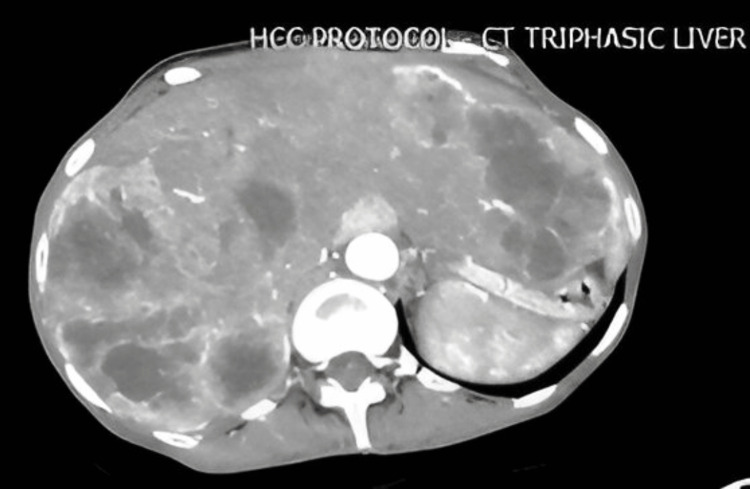
CT scan shows multiple large heterogenous and necrotic lesions in both lobes of liver. CT, computed tomography; HCC, hepatocellular carcinoma

**Figure 2:**
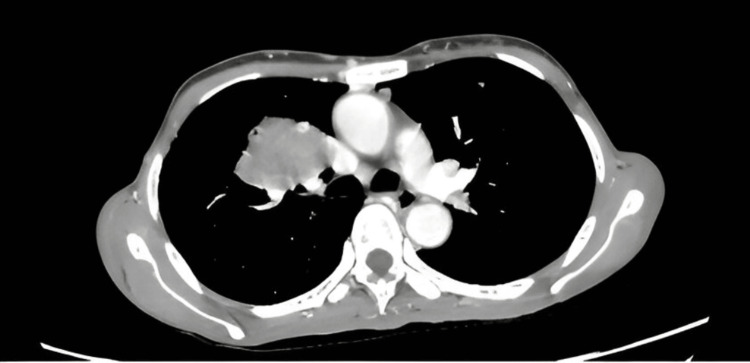
CT scan shows multiple rounded lesions with spiculated margins in bilateral lungs. CT, computed tomography

A CT scan triphasic liver with hepatocellular carcinoma (HCC) protocol and CT chest with contrast were performed at PKLI, which revealed multifocal bilobar hepatic lesions (Figure 1) showing central necrosis, along with innumerable rounded bilateral lung lesions (Figure 2). Metastatic posterior mediastinal and right mid-jugular lymph node masses were also observed. These findings were suggestive of either multiple lung and hepatic metastases from an unknown primary or a primary lung malignancy with pulmonary and hepatic metastases. Subsequently, a biopsy of the liver lesion was performed and submitted to the Histopathology Department for evaluation.

Gross and microscopic examination

**Figure 3:**
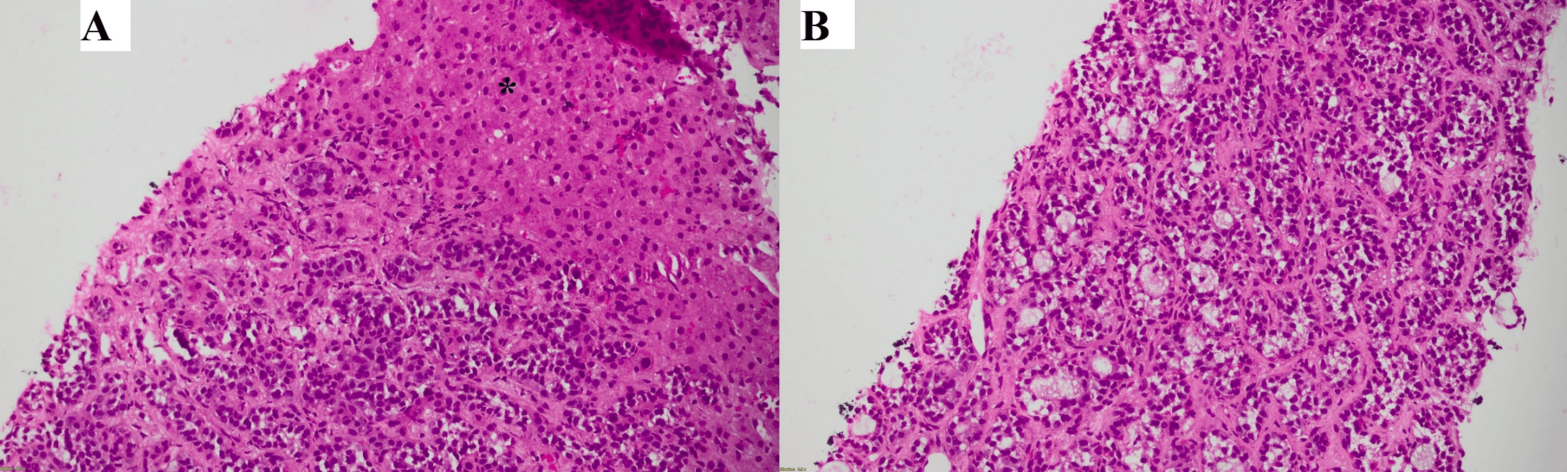
(A) Low-power view of the liver core showing benign parenchyma (asterisk*) with an adjacent area showing a neoplasm. (B) Low-power view of neoplastic cells arranged in nests, forming microcystic spaces and a cribriform pattern.

**Figure 4:**
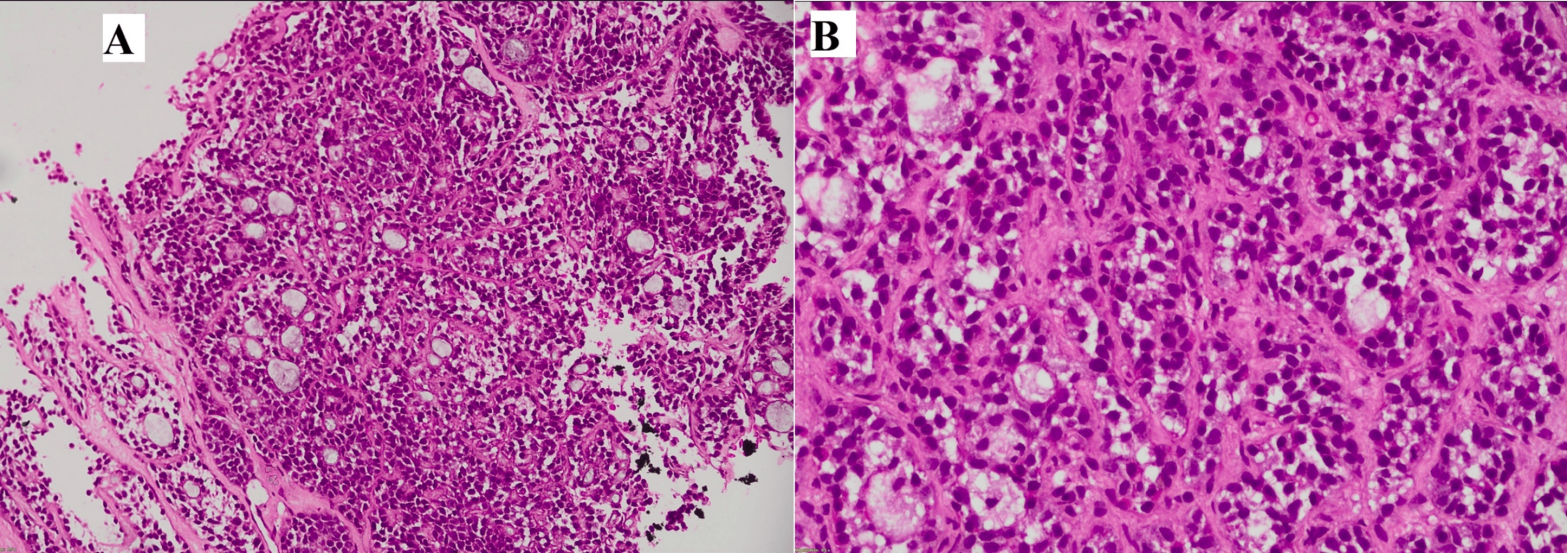
(A-B) High-power view of neoplastic cells with hyperchromatic nuclei, surrounding gland-like spaces filled with eosinophilic myxoid material.

**Figure 5:**
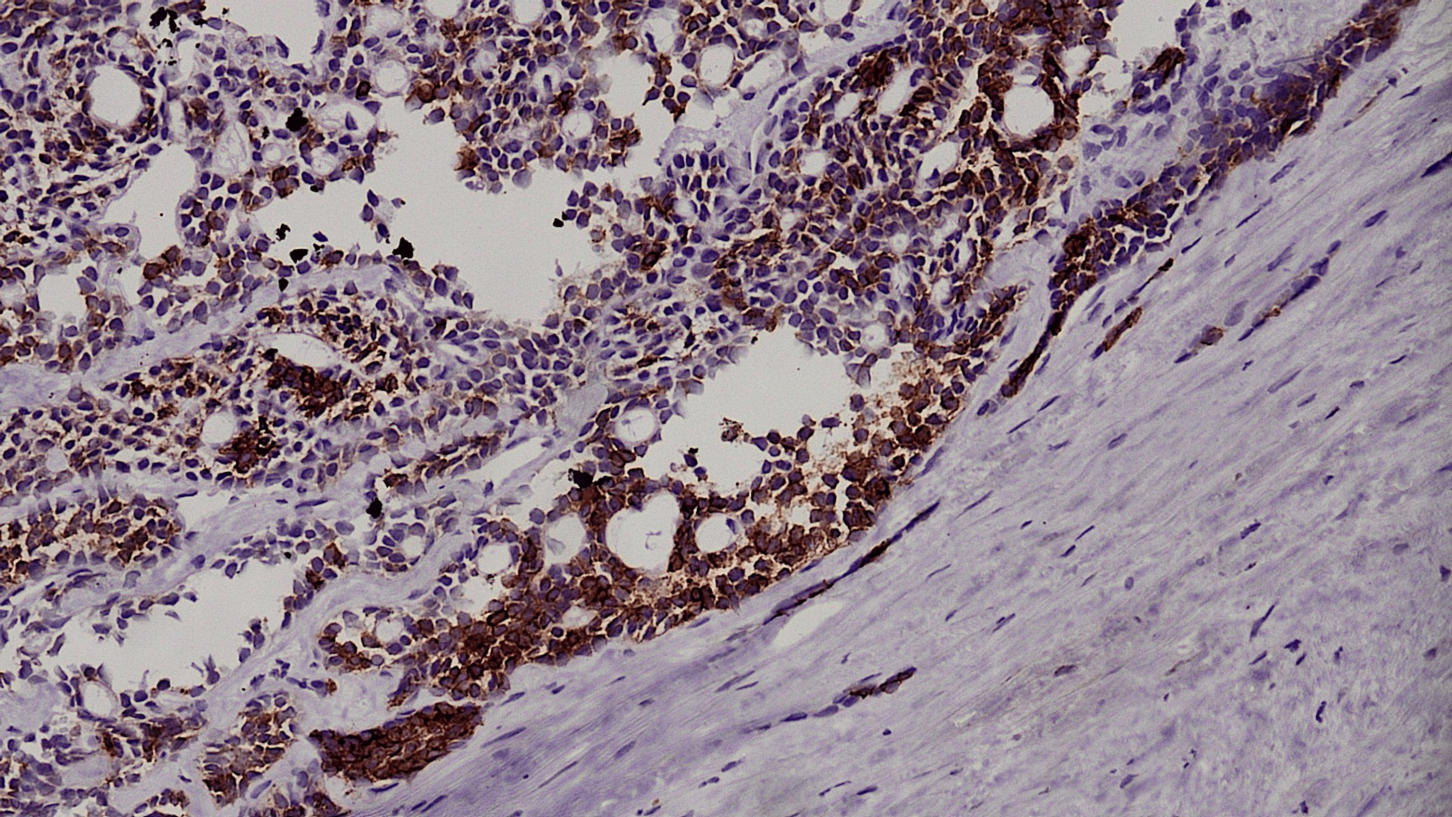
CD117 IHC diffuse positive in neoplastic cells. IHC, immunohistochemistry

**Figure 6:**
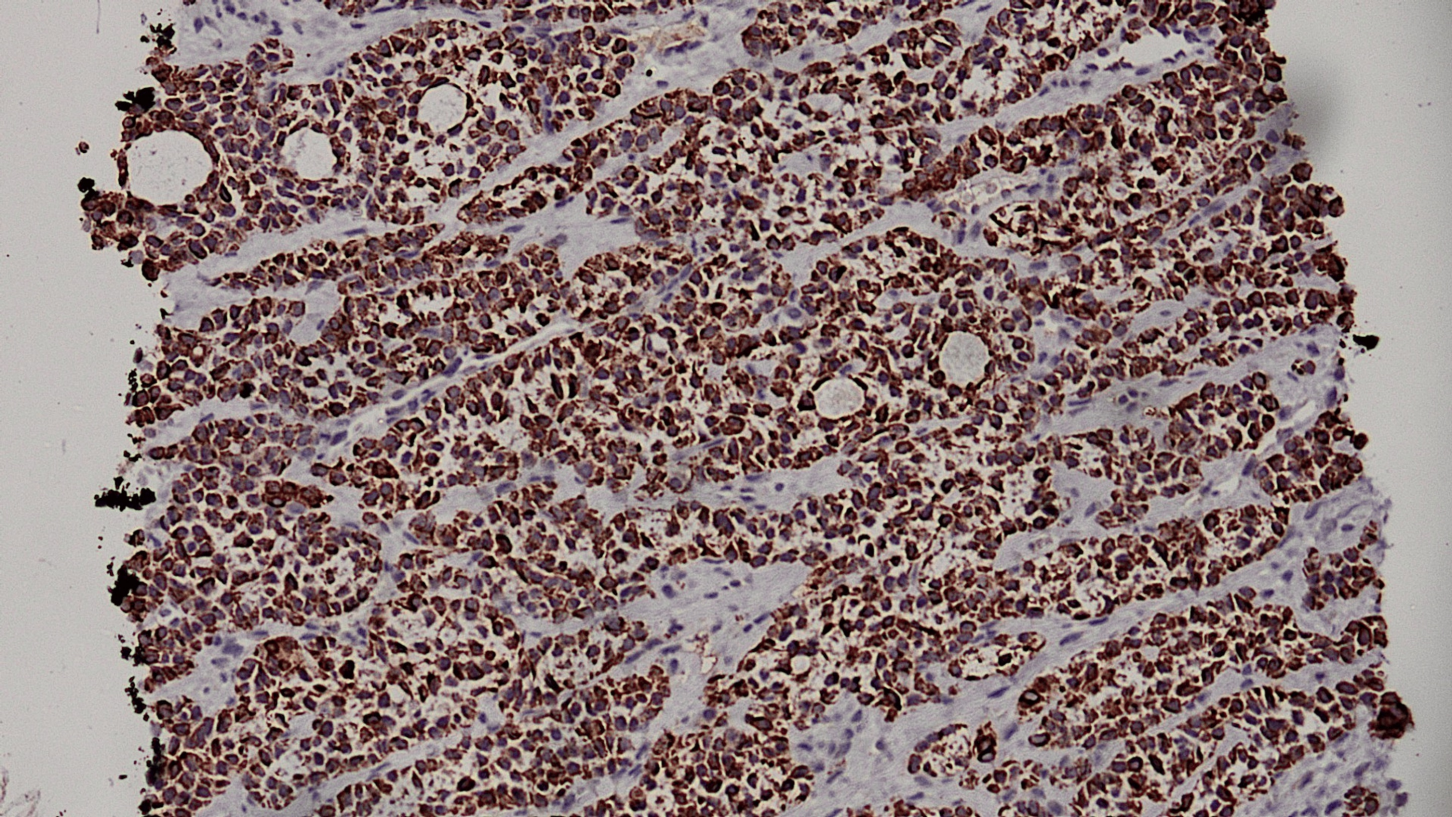
CK7 IHC diffuse positive in neoplastic cells. IHC, immunohistochemistry

**Figure 7:**
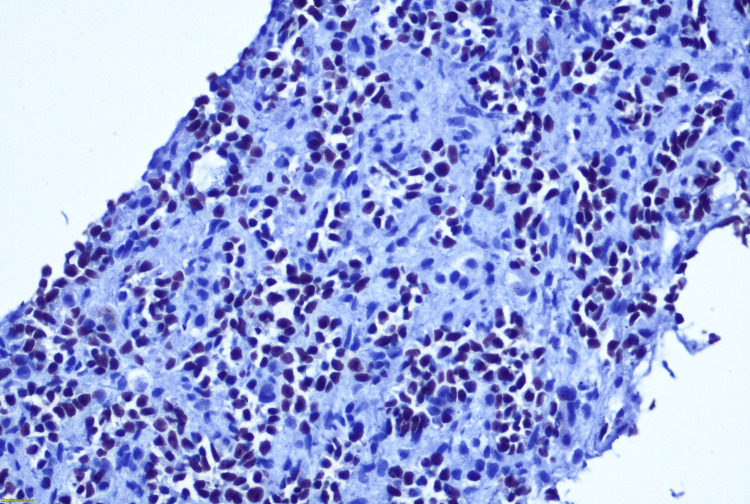
P63 IHC diffuse positive in neoplastic cells. IHC, immunohistochemistry

**Figure 8:**
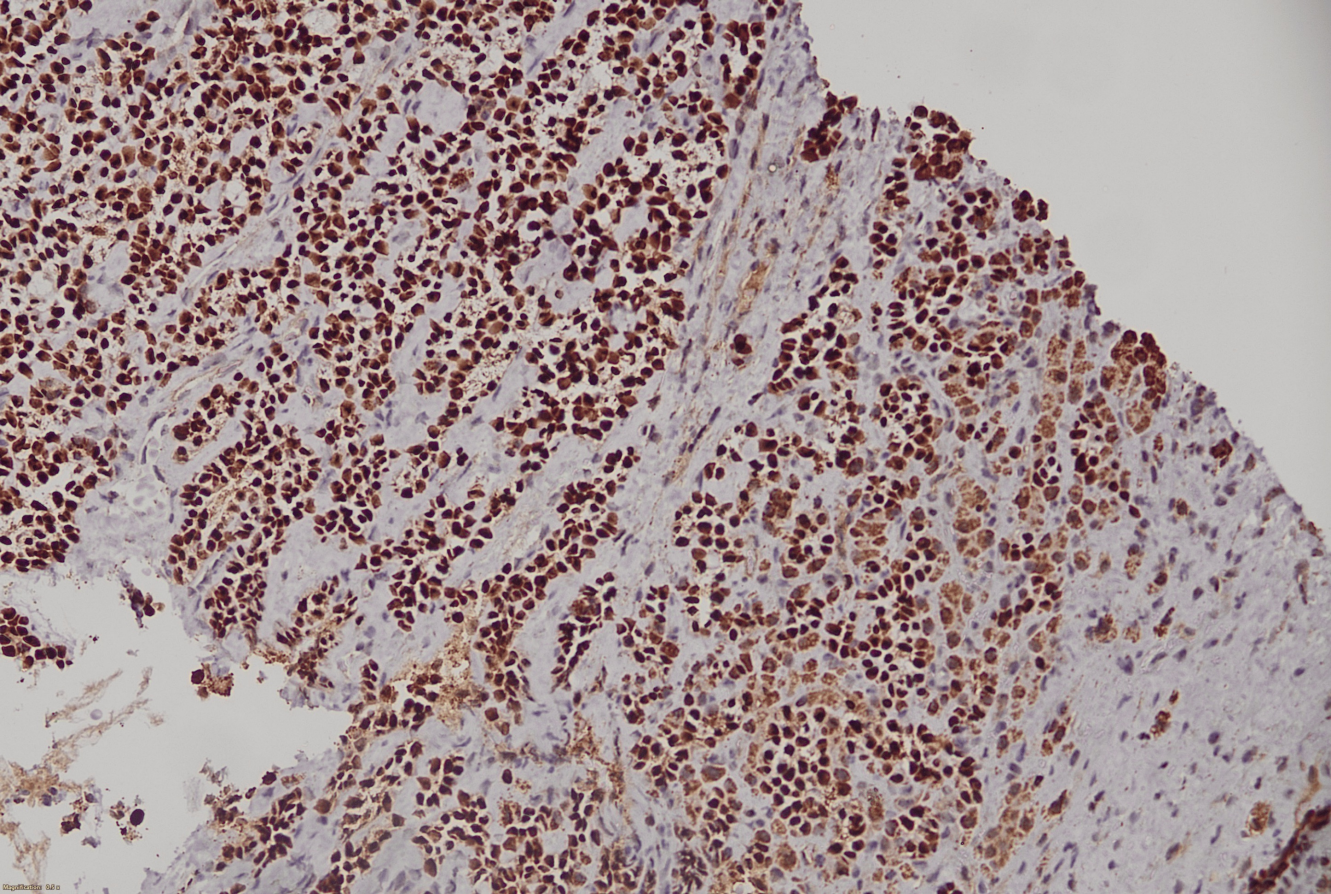
SOX-10 IHC diffuse positive in neoplastic cells. IHC, immunohistochemistry

Two cores of liver parenchyma, one measuring 1.3 cm and the other 1.8 cm in length, were sent for histopathology. Microscopic examination revealed liver parenchyma showing infiltration by an epithelial neoplasm composed of nests of tumor cells with microcystic spaces, often exhibiting a cribriform pattern (Figure 3). Tumor cells showed basaloid morphology, with hyperchromatic, round-to-oval angulated nuclei and scant cytoplasm. The cystic spaces were filled with eosinophilic to amphophilic material (Figure 4). A panel of immunohistochemical stains was applied, which showed diffuse positivity for CK7, P63, SOX-10, and CD117 (Figures 5-8).

## Discussion

Hepatic metastasis of ACC is very rare. Currently, there is limited published clinical and radiologic data describing the features of ACC metastasizing to the liver [3,4]. Due to the long clinical course of this tumor, distant metastasis is often a late presentation [3,5]. In our literature search, a case was reported of a 54-year-old female presenting with a solitary hepatic lesion 30 years after being diagnosed with ACC of the submandibular gland [5]. Our patient presented with hepatic and lung lesions after a 10-year gap from her initial submandibular swelling (FNAC had been performed at the time and was diagnosed as a benign salivary gland neoplasm).

Another interesting fact is the synchronous presentation of late metastatic ACC [6], as was the case in our patient. She had multiple metastatic lesions in the lungs and liver. On histopathology, solid and cribriform patterns are associated with more biologically aggressive behavior [6,7]. In our case, the tumor showed a cribriform pattern, which is considered an aggressive variant. 

There are multiple management options for metastatic ACC, including chemoembolization, radiofrequency ablation, and surgical resection. Unfortunately, the role of each modality is questionable [8]. There is no FDA-approved treatment modality for metastatic ACC [9]. A few systemic therapies are available, such as cytotoxic agents and multikinase inhibitors targeting the vascular endothelial growth factor receptor, but these therapies have modest activity [10].

Metastatic ACC to the liver carries a poor prognosis. A retrospective single-center cohort and literature study (2000-2018) found that liver metastases from ACC carry a poor prognosis, with a median survival of only 14 months and a three-year survival rate of around 15% [11]. These tumors are usually resistant to chemotherapy, as reported in a case by Balducci et al. [2]. Our patient was referred to the oncologist, who started her on chemotherapy; unfortunately, she did not respond well and expired in July 2025, almost 10 months after the initial diagnosis.

## Conclusions

Some lessons to take away from this report are the importance of early diagnosis of ACC and the role of histopathology in this regard. Delayed diagnosis can have long-term consequences; as was the case in this report, our patient had a submandibular swelling for nearly a decade but did not seek medical attention until she developed abdominal pain from liver metastasis. This underscores the need for public awareness and early evaluation of persistent, painless head and neck swellings, which may represent indolent malignancies like ACC.
